# A model for predicting return of spontaneous circulation and neurological outcomes in adults after in-hospital cardiac arrest: development and evaluation

**DOI:** 10.3389/fneur.2023.1323721

**Published:** 2023-11-17

**Authors:** Zheng Li, Jihong Xing

**Affiliations:** Department of Emergency Medicine, The First Hospital of Jilin University, Changchun, Jilin, China

**Keywords:** cardiopulmonary resuscitation, in-hospital cardiac arrest, neurological function, prognostic model, return of spontaneous circulation

## Abstract

**Introduction:**

In-hospital CA (IHCA) is associated with rates of high incidence, low return of spontaneous circulation (ROSC), low survival to discharge, and poor neurological outcomes. We aimed to construct and evaluate prediction models for non-return of spontaneous circulation (non-ROSC) and poor neurological outcomes 12 months after ROSC (PNO-12).

**Methods:**

We retrospectively analyzed baseline and clinical data from patients experiencing cardiac arrest (CA) in a big academic hospital of Jilin University in China. Patients experiencing CA between September 1, 2019 and December 31, 2020 were categorized into the ROSC and non-ROSC groups. Patients maintaining ROSC >20 min were divided into the good and PNO-12 subgroups.

**Results:**

Univariate and multivariate logistic regression identified independent factors associated with non-ROSC and PNO-12. Two nomogram prediction models were constructed and evaluated. Of 2,129 patients with IHCA, 851 were included in the study. Multivariate logistic regression analysis revealed that male sex, age >80 years, CPR duration >23 min, and total dose of adrenaline >3 mg were significant risk factors for non-ROSC. Before CA, combined arrhythmia, initial defibrillation rhythm, and advanced airway management (mainly as endotracheal intubation) also influenced outcomes. The area under the receiver operating characteristic curve in the prediction model was 0.904 (C-index: 0.901). Respiratory failure, shock, CA in the monitoring area, advanced airway management, and noradrenaline administration were independent risk factors for PNO-12. The AUC was 0.912 (C-index: 0.918).

**Conclusions:**

Prediction models based on IHCA data could be helpful to reduce mortality rates and improve prognosis.

## 1 Introduction

In-hospital cardiac arrest (IHCA) is a common event facing emergency physicians worldwide. High-quality cardiopulmonary resuscitation (CPR) is crucial to improve patient outcomes. Two key prognostic indicators in IHCA cases are the return of spontaneous circulation (ROSC) and a good neurological prognosis. Unfortunately, IHCA is associated with rates of high incidence, low ROSC, low survival to discharge, and poor neurological outcomes. The financial impact of prolonging life must be considered in countries with high healthcare costs. Therefore, the impact of IHCA on quality of life is paramount worldwide.

A clinical prognosis prediction model should be developed to rapidly and efficiently evaluate the disease and predict outcomes to address this issue. Although various prognostic models have been introduced since 1989, such as the PAM score (1), the CASPRI score (2), the RSSR score (3), and the GO FAR2 score (4), most of these scores focus primarily on parameters related to the acute disease. However, they ignore the important factors associated with chronic disease, limiting their use for accurate diagnosis, treatment, and prognostic evaluation. This study adopts the international Utstein model of the CPR effect evaluation model and collects data to construct short- and long-term prognostic prediction models. Evaluation results showed that the models can help clinicians provide more suitable treatment and predict the prognosis for adult patients with IHCA.

## 2 Materials and methods

This study used the hospital's digital record system, outpatient medical records, and medical information related to CPR to retrieve data from September 1, 2019, to December 31, 2020. This study included adult patients (aged 18 years and older) who underwent IHCA in various departments including the general ward, emergency room, intensive care unit, operating room, and catheterization room. Patients with no-first CA, incomplete clinical data, congenital heart disease, during pregnancy or perinatal period, and with severe trauma were excluded from the study. We tracked and organized their baseline and clinical data. Based on CPR outcome, patients were categorized into either the ROSC (maintaining ROSC for ≥20 min) or non-ROSC group, which was the primary outcome.

We followed the ROSC group to assess their neurological prognostic status at 12 months mainly by phone interview with the participants or their families. They were divided into two groups by cerebral performance category (CPC), and the detailed evaluation criteria are presented in Table 1 (5).

**Table 1:** Cerebral performance category (5).

**CPC level**	**Illustrations**
CPC 1	Good cerebral performance: conscious, alert, able to work, might have mild neurologic or psychologic deficit
CPC 2	Moderate cerebral disability: conscious, sufficient cerebral function for independent activities of daily life. Able to work in sheltered environment
CPC 3	Severe cerebral disability: conscious, dependent on others for daily support because of impaired brain function. Ranges from ambulatory state to severe dementia or paralysis
CPC 4	Coma or vegetative state: any degree of coma without the presence of all brain death criteria. Unawareness, even if appears awake (vegetative state) without interaction with environment; may have spontaneous eye opening and sleep/awake cycles. Cerebral unresponsiveness
CPC 5	Brain death: apnea, areflexia, EEG silence, etc.

Participants with a CPC 1–2 status were further categorized into the good neurological outcome group and the others with CPC 3–5 status were categorized into the poor neurological outcome group (PNO-12). PNO-12 was the secondary outcome.

All data were statistically analyzed using SPSS 26.0 or R 4.2. Continuous variables are represented as mean ± standard deviation or median with interquartile spacing (IQR). The normality of the data was based on their distribution. Student's *t*-test was used for normally distributed data, while the Mann–Whitney *U*-test was used for nonnormally distributed data. Count data are expressed as frequency (%), and the chi-square test or Fisher's exact probability method was used for group comparison based on theoretical frequency. A univariate logistic regression analysis was performed for continuous variables, with non-ROSC and PNO-12 as the dependent variable (*Y*). A receiver operating characteristic (ROC) curve was plotted to determine the threshold of the dependent variable using the maximum Youden index method. The dependent variable was then converted into a dichotomous variable (0 or 1). Univariate logistic regression analysis was performed with the variables related to Non-ROSC and PNO-12. Variables with a *P* < 0.1 were included in the multivariate logistic regression model to identify independent influencing factors. Multiple regression models were analyzed using dual variables, and a nomogram prediction model was constructed based on the results. Using bootstrap resampling, the AUC was calculated to evaluate, and a calibration curve was plotted. The C-index and the mean absolute error (MAE) were calculated, and the Hosmer–Lemeshow goodness-of-fit test (H–L test) was performed to evaluate the calibration degree of the model. Additionally, the decision curve analysis method and clinical impact curves were used to assess the clinical applicability of the prediction model. External validation was performed using data from another big academic hospital in Jilin University. The statistical method had been verified by the Administrative Committee for Clinical Research of the First Hospital of Jilin University (num.2023-KS-223).

### 2.1 Ethics statement

The study was conducted in accordance with the Declaration of Helsinki and approved by the Institutional Review Board of the First Hospital of Jilin University for studies involving humans (Date 2023/09/27, approval num. 2023-649). Informed consent was waived by the board.

## 3 Results

**Figure 1:**
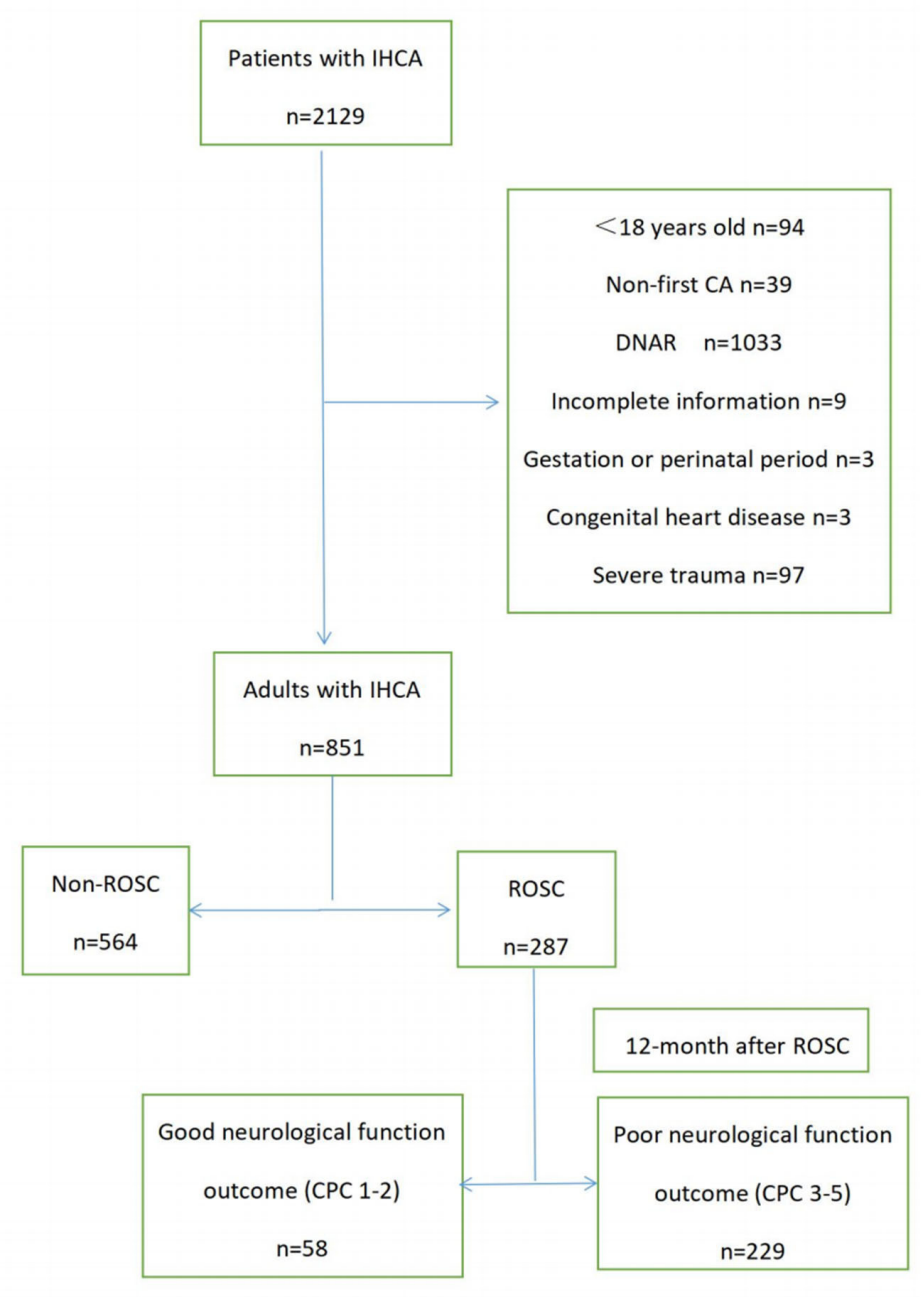
Flowchart of the patient selection.

During the study period, 2,129 patients experienced IHCA. Based on the study criteria, 851 patients were finally included (Figure 1). A total of 564 (66.27%) patients achieved ROSC, and of these, 229 (26.91%) were categorized as PNO-12 (Supplementary Table 1).

### 3.1 Development of the predictive model for the primary outcome

**Figure 2:**
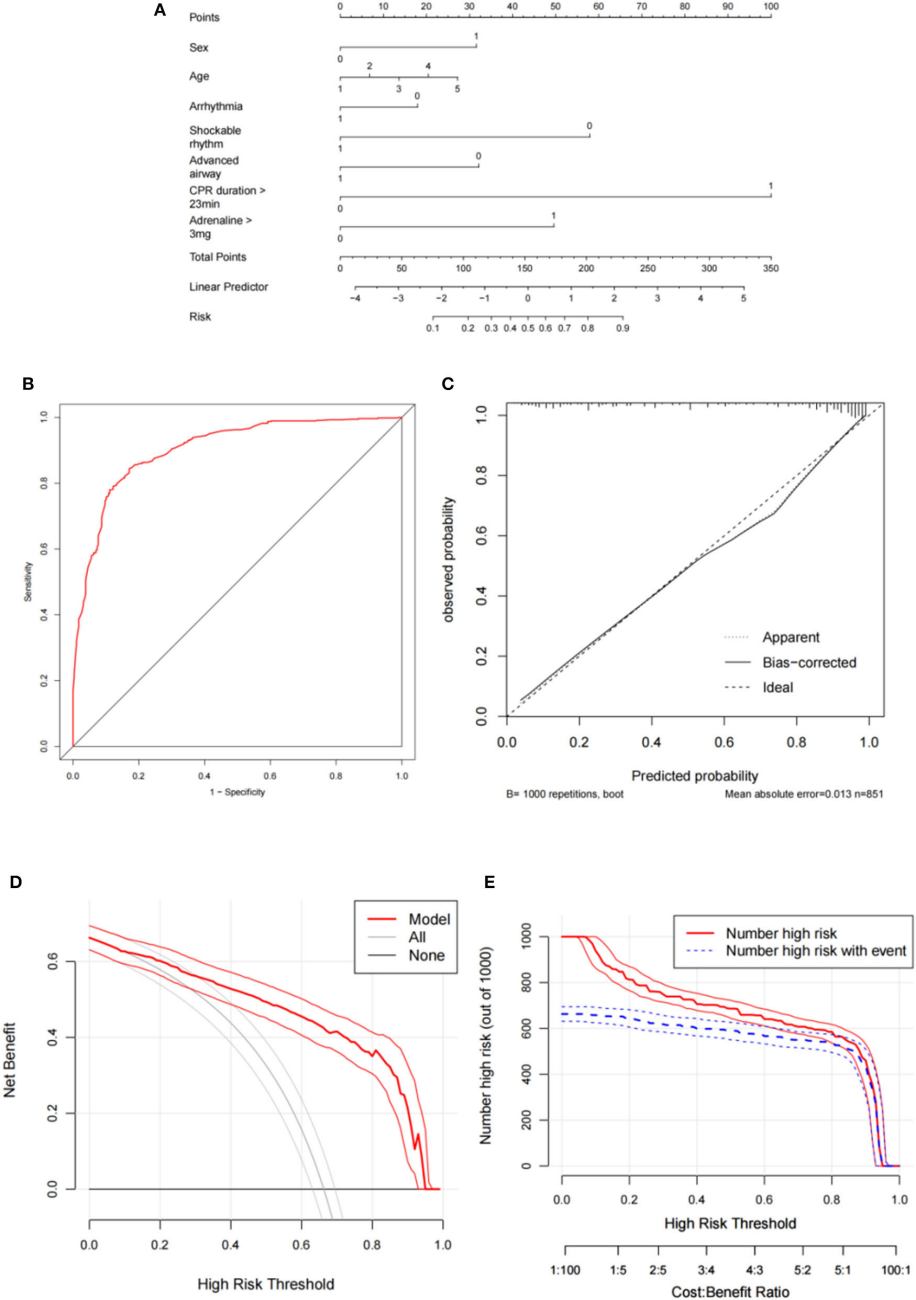
Development and evaluation of the predictive model for primary outcome. **(A)** Nomogram; **(B)** Receiver operating curve; **(C)** Calibration curve; **(D)** Decision curve analysis; **(E)** Clinical impact curve. CPR, cardiopulmonary resuscitation.

Supplementary Table 1 compares the baseline and clinical data between the ROSC and non-ROSC groups. Table 2 shows the dichotomized thresholds for the duration of CPR (CD) and the total dose of adrenaline (TDOA) using the ROC analysis of univariate logistic regression by primary/secondary outcome. All variables with *P* < 0.1 in the results of the univariate logistic regression were included in the multivariate logistic regression analysis (Table 3). The results indicate that male sex [odds ratio (OR) = 2.42, 95% CI: 1.58–3.69, *P* < 0.001], age >80 years (OR = 3.31, 95% CI: 1.57–6.97, *P* = 0.002), CD >23 min (OR = 19.18, 95% CI: 12.01–30.63, *P* < 0.001), and TDOA >3 mg were independent risk factors for non-ROSC. Before CA, arrhythmia (OR = 3.98, 95% CI: 2.43–6.51, *P* < 0.001), an initial defibrillation heart rhythm (OR = 0.20, 95% CI: 0.11–0.35, *P* < 0.001), and advanced airway management (OR = 0.45, 95% CI: 0.29–0.71, *P* = 0.001) were identified as independent protective factors (Table 3). The predictive model was constructed using R 4.2 (Figure 2A).

**Table 2:** Dichotomized thresholds of CPR duration and total dosage of adrenaline using receiver operating characteristic analysis of univariate logistic regression by primary/secondary outcome.

	**Cut-off**	**Sensitivity**	**Specificity**	**AUC**
CD, min	23.5^†^/9.5^‡^	0.830^†^/0.546^‡^	0.805^†^/0.759^‡^	0.877^†^/0.654^‡^
TDOA, mg	3.5^†^/0.5^‡^	0.573^†^/0.686^‡^	0.882^†^/0.776^‡^	0.785^†^/0.760^‡^

AUC, area under the receiver operating characteristic curve; CD, CPR duration; TDOA, total dosage of adrenaline.

^†^Primary outcome.

^‡^Secondary outcome.

**Table 3:** Multivariate logistic regression analysis results by primary and secondary outcomes.

**Categories**	**Regression coefficient**	**Standard error**	***P-*value**	**OR (95%CI)**
Primary outcome^†^				
Male	0.88	0.22	<0.001	2.42 (1.58–3.69)
≥80 years	1.20	0.38	0.002	3.31 (1.57–6.97)
Arrhythmia	−0.50	0.25	0.041	0.61 (0.38–0.98)
Defibrillation rhythm	−1.62	0.28	<0.001	0.20 (0.11–0.35)
CD >23 min	2.95	0.24	<0.001	19.18 (12.01–30.63)
AAM	−0.80	0.23	0.001	0.45 (0.29–0.71)
TDOA >3 mg^a^	1.38	0.25	<0.001	3.98 (2.43–6.51)
Secondary outcome^‡^				
Respiratory-F	1.91	0.83	0.021	6.73 (1.33–34.07)
Shock	1.59	0.63	0.008	4.91 (1.51–15.99)
Intensive care	1.49	0.46	0.001	4.42 (1.80–10.88)
Cardiac etiology	−2.00	0.55	<0.001	0.14 (0.05–0.40)
Defibrillation rhythm	−0.95	0.44	0.031	0.39 (0.16–0.92)
AAM	1.23	0.52	0.018	3.43 (1.24–9.51)
Norepinephrine	1.16	0.51	0.023	3.19 (1.17–8.66)

OR, odds ratio; CI, confidence interval; CD, CPR duration; AAM, advanced airway management; TDOA, total dosage of adrenaline; Respiratory-F, respiratory failure.

^†^Primary outcome.

^‡^Secondary outcome.

^a^Dichotomized thresholds.

### 3.2 Evaluation and validation of the predictive model for the primary outcome

The discriminative performance of the nomogram model was evaluated by plotting the ROC curves, resulting in an AUC of 0.904 (95% CI: 0.882–0.925), indicating good model discrimination (Figure 2B). Internal validation was conducted using the bootstrap method, which involved random sampling performed 1,000 times. The C index was 0.901 (95% CI: 0.879–0.922) with an MAE of 0.013. Furthermore, the H–L test (*X*^2^ =12.36, *P* = 0.136) indicated a good fit and calibration of the model (Figure 2C). The nomogram prediction model demonstrated good clinical applicability, as evidenced by decision curve analysis and clinical impact curves (Figures 2D, E). The external validation, which included 355 participants showed an AUC of 0.900 (95% CI: 0.868–0.933), and the calibration curve showed good consistency (**Figures 4A, B**).

### 3.3 Development of a predictive model for the secondary outcome

Supplementary Table 1 compares the baseline and clinical data of the groups with good neurological outcomes and PNO-12. Variables with *P* < 0.1 in the univariate logistic regression analysis were included in the multivariate logistic regression analysis. The results showed that respiratory failure (OR = 6.73, 95% CI: 1.33–34.07, *P* = 0.021), shock (OR = 4.91, 95% CI: 1.51–15.99, *P* = 0.008), and cardiac arrest (CA) occurring in the monitoring area (OR = 4.42, 95% CI: 1.80–10.88, *P* = 0.001); advanced airway management (OR = 3.43, 95% CI: 1.24–9.51, *P* = 0.018); and the use of noradrenaline (OR = 3.19, 95% CI: 1.17–8.66, *P* = 0.023) were independent risk factors for PNO-12.

**Figure 3:**
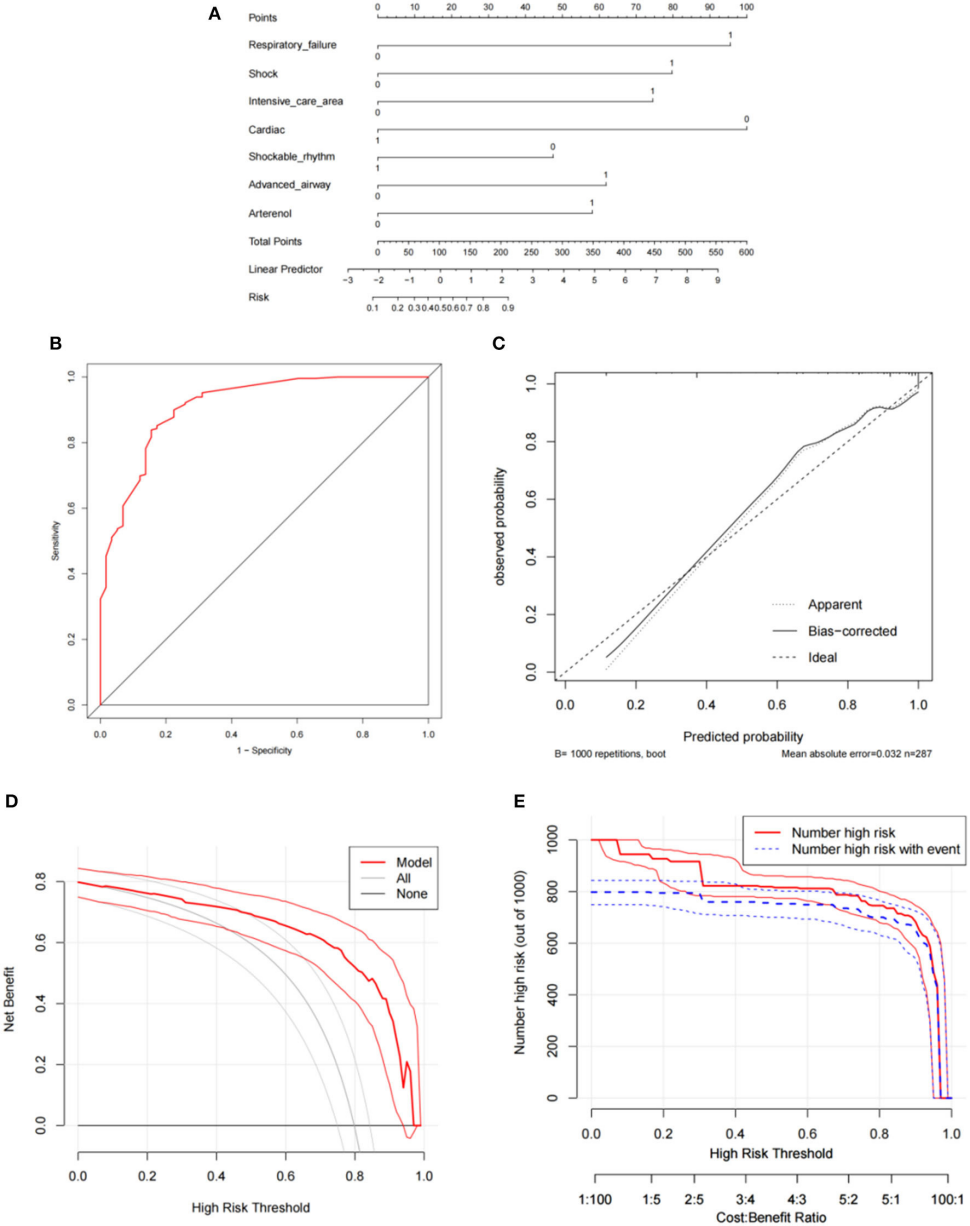
Development and evaluation of the predictive model for secondary outcome. **(A)** Nomogram; **(B)** Receiver operating curve; **(C)** Calibration curve; **(D)** Decision curve analysis; **(E)** Clinical impact curve. CPR, cardiopulmonary resuscitation.

Furthermore, cardiac etiology (OR = 0.14, 95% CI: 0.05–0.40, *P* < 0.001) and an initial defibrillation rhythm (OR = 0.39, 95% CI: 0.16–0.92, *P* = 0.031) were identified as independent protective factors (Table 3). The predictive model is shown in Figure 3A.

### 3.4 Evaluation and validation of the predictive model for the secondary outcome

**Figure 4:**
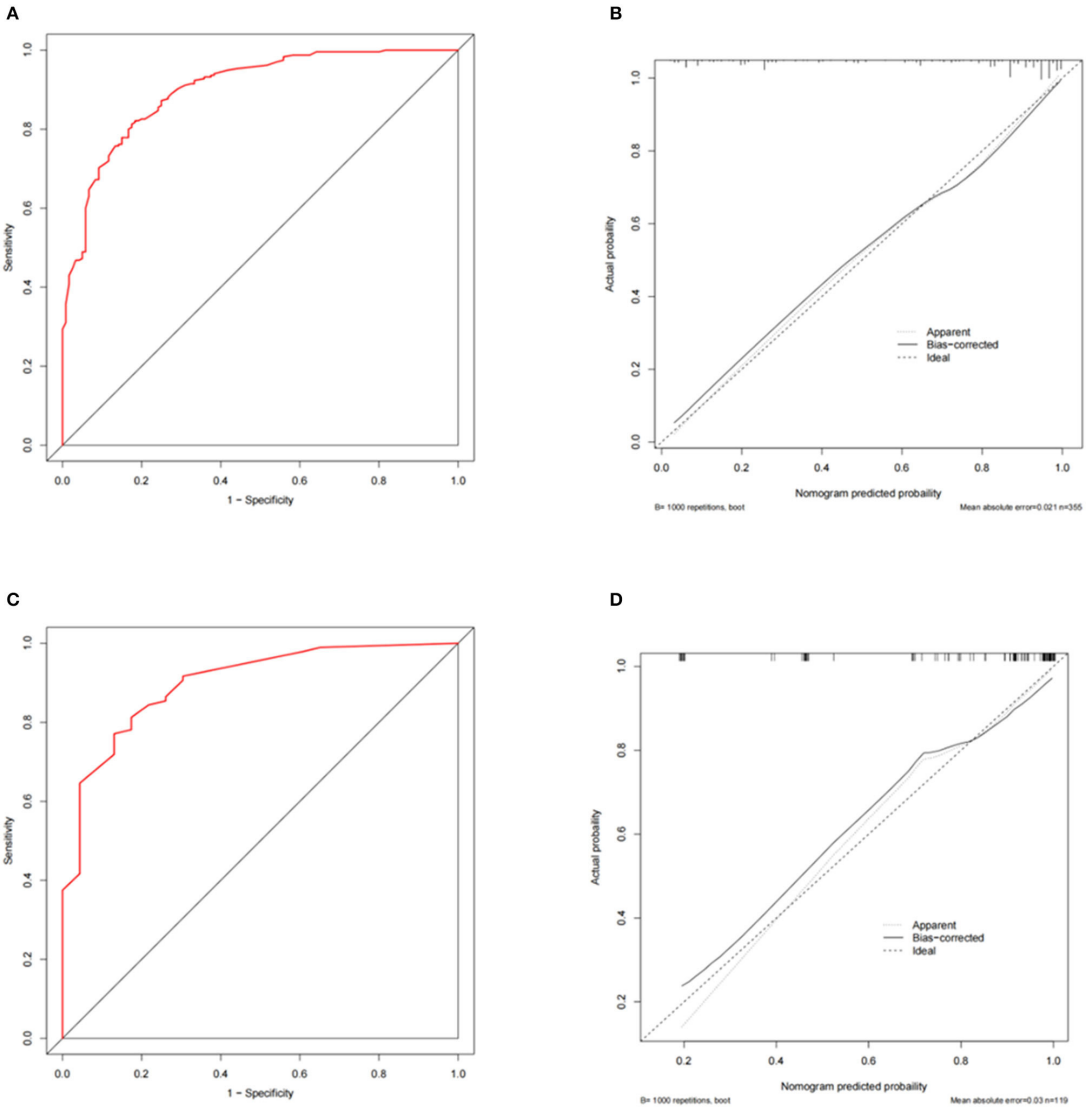
External validation of the predictive models for primary and secondary outcomes. **(A, B)** Receiver operating curve and calibration curve for primary outcome; **(C, D)** Receiver operating curve and calibration curve for secondary outcome.

The AUC for the nomogram model was 0.912 (95% CI: 0.870–0.954), indicating a good discriminative performance for the model (Figure 3B). The C index was 0.918 (95% CI: 0.875–0.957), and the MAE was 0.032. The H–L test (*X*^2^ =11.10, *P*=.196) indicated the model was well calibrated (Figure 3C). The prediction model demonstrated good clinical applicability, as evidenced by decision curve analysis and clinical impact curves (Figures 3D, E). The result of the external validation, which included 120 participants, showed an AUC of 0.909 (95% CI: 0.833–0.985), and the calibration curve also showed good consistency (Figures 4C, D).

## 4 Discussion

In this study, 851 patients with IHCA were included, predominantly consisting of males (543, 63.81%). The median age was 65 (54–74) years. A comprehensive review conducted in the US (6) also reported a mean age of 66 years for patients with IHCA and that 58% of patients with IHCA were male. The ROSC rate in our study was 33.73%, slightly lower than the 35.5% rate reported in a previous study conducted in Beijing (7). The disparity in the level of regional medical development may be a plausible reason for this difference. Male sex and advanced age were identified as independent risk factors for non-ROSC. However, the relationship between sex and PNO-12 was not significant, which is consistent with the results of previous studies (8). Nevertheless, several studies (9) show that advanced age is a risk factor for poor prognosis and even death among patients with an IHCA. This may be due to the decline in physiological function and immunity among older adults or various underlying diseases that make them highly sensitive to the pathophysiological state of ischemia and hypoxia in CA, resulting in poorer tolerance. Additionally, older family members are less likely to receive active treatment due to the influence of traditional ethics, contributing to the poor prognosis of IHCA among older patients in this study.

Respiratory failure and shock before the IHCA were independent factors influencing PNO-12. Both conditions represent the advanced stage of disease progression, indicating the severe condition of the patients. Furthermore, based on the analysis of clinical data from 40,000 patients, Chan et al. (2) concluded that hypotension was a factor contributing to the failure of CPR. Among the 222 patients with arrhythmia before IHCA, 63.96% had a cardiac etiology, suggesting that it may be an independent factor in predicting non-ROSC.

The etiology of IHCA etiology is usually recorded in the Utstein mode based on clinical data and is classified into cardiac and non-cardiac causes. A previous review (6) inferred that unexplained CA is of cardiac origin. In this study, it was determined to be caused by lesions of the heart itself and excluded by definite non-cardiac causes, introducing a degree of subjective bias. The primary cause of IHCA was mainly non-cardiac (58.40%).

A 3-year retrospective study (10) in Denmark demonstrated that patients with CA with a cardiac etiology had a better prognosis rate for neurological outcomes than those with a non-cardiac etiology. Another study (11) found that patients with acute myocardial infarction had a higher survival rate than non-AMI patients. This difference may be attributed to recent advances in coronary intervention technology, including prompt treatment, physical exercise, regular patient monitoring, and comprehensive management of chronic diseases, significantly reducing the incidence of CA.

The location of the IHCA called the “monitoring area,” was identified as an independent protective factor that predicted PNO-12. The traditional perception is that patients in a monitoring area have a better prognosis than those in general wards because of the resuscitation experience of medical and nursing personnel and the availability of advanced comprehensive emergency equipment. However, the opposing view is that patients in a “monitoring area” are more severely ill, leading to poor prognosis, even with high-quality CPR.

The initial rhythm type of IHCA can be divided into defibrillation and non-defibrillation rhythms. Our results revealed that patients with a defibrillation rhythm had better neurological prognoses than patients with a non-defibrillation rhythm in both predictive models. This finding is consistent with several studies; Hirlekar et al. (12) and Aufderheide et al. (13) also found that patients in CA with non-shockable rhythms have favorable neurological prognoses, ranging from 2 to 15%.

A cohort study involving 114,628 patients with a non-shockable rhythm OHCA (9) demonstrated a significant association between reverting to a shockable rhythm after cycles of CPR and good functional prognoses. Studies conducted in China and abroad have consistently shown that patients with a series of defibrillator shocks during CA exhibit higher rates of ROSC. For acutely ill patients at risk of developing IHCA, monitoring the cardiac rhythm (ECG) and administering appropriate medication and other treatments to reduce mortality rates and improve prognosis is important.

In this study, endotracheal intubation (ETI) was the predominant method of establishing an advanced airway, positively affecting ROSC in patients with IHCA. However, it may also negatively impact neurological outcomes. Brain tissue is highly sensitive to ischemia and hypoxia. Therefore, an advanced airway can fulfill the oxygen demand and improve the metabolic state of the body, particularly the heart and brain tissue, to increase the rate of ROSC. Compared to a balloon mask, an advanced airway can reduce the risk of aspiration to a certain extent.

A study by Aufderheide et al. (13) in prehospital emergency patients showed that establishing an advanced airway was associated with poor long-term neurological outcomes. This may be attributed to unskilled ETI procedures, interrupted chest compression, or inappropriate ventilation. On the contrary, Yeung et al. (10) showed that ETI in patients with IHCA took only 15.8 s and had a high success rate. Furthermore, the decision to perform ETI in patients with IHCA should be made by experienced clinicians to minimize or avoid potential damage caused by the procedures. The benefit of ETI may be greater in patients with a non-defibrillation rhythm than in patients with a defibrillation rhythm (11).

There is no consensus on the optimal time to establish an advanced airway. A single-center retrospective study involving 702 patients in China (14) reported a mean ETI time of 8.8 min, negatively associated with a good neurological prognosis. In contrast, a study found that patients who underwent ETI within 15 min before CPR had worse outcomes than patients who did not (10).

Previous studies consistently show that ROSC and survival rates decrease with increasing CD (15, 16). In this study, the non-ROSC and PNO-12 groups had longer CD (9 min vs. 45 min, *P* < 0.001, respectively; 4.5 min vs. 10 min, *P* < .001, respectively), and these differences were significant. The final results revealed that CD > 23 min independently influenced non-ROSC and was associated with poor prognosis. Timely, continuous, and effective chest compression can improve the ROSC rate. However, prolonged CD leads to irreversible heart, brain, and lung damage caused by ischemia and reperfusion.

Furthermore, prolonged fatigue during chest compression significantly reduces the quality of CPR and negatively affects outcomes. Currently, there is no consensus on the optimal CD. In China, a CD time of 30 min is mostly used as the standard definition. In comparison, the 2015 European Resuscitation Council guidelines recommend discontinuing CPR for cases of irreversible etiology after 20 min (17). However, the optimal CD for other cases remains unclear. Recently, successful reports following long CD (>30 min) have emerged, raising the possibility that CD indirectly reflects the severity of the condition and the quality of CPR (18).

Considering individual patient differences and conditions, further multi-center and large-scale clinical studies are necessary to categorize CA-related factors to define the “best threshold” at all levels.

The latest American Heart Association and European Resuscitation Council guidelines recommend the early use of adrenaline, with higher levels of evidence and recommendation (19, 20). Adrenaline strengthens left ventricular and cerebral blood perfusion by exciting α-1 receptors and increasing aortic diastolic pressure, thus improving ROSC and survival rates in patients with CA. However, higher adrenaline (DOA) doses may promote subendocardial vasoconstriction, thus increasing the risk of malignant arrhythmia. With longer CD, increased DOA also leads to a poor neurological prognosis (21). This effect could be due to the simultaneous excitation of the β1 receptor, leading to excessive myocardial oxygen consumption and increased PAMD.

Adrenaline was administered in 662 cases with a median total dose of 3 (IQR: 1, 7) mg in this study. The study findings indicated that TDOA >3 mg was an independent risk factor for non-ROSC, although it could be insignificant for PNO-12. The disparity in the results may be attributed to several reasons. First, this study was conducted at a single center with a small sample size, which limited the demonstration of the efficacy of adrenaline administration. Second, the study targeted adult patients with IHCA, while similar studies also included patients with OHCA or overall CA.

Recently, some studies (9) used the adrenaline administration rate (total dose/CPR length, mg/min) as an indicator of adrenaline application, reporting that a higher adrenaline administration rate may be associated with a poor neurological outcome after CPR. Furthermore, among IHCA patients who received the same DOA, patients with a body weight of 82.5 kg had a worse prognosis than those with lower body weight. Furthermore, Soar et al. (22) showed that adrenaline better treats patients whose initial heart rhythm is non-shockable.

Noradrenaline is a typical α-receptor agonist, and administration increases aortic and diastolic pressure. In this study, noradrenaline emerged as an independent influencing factor for PNO-12. Callaham et al. (23) reported similar findings in a study showing that noradrenaline had no positive effect on the prognosis of patients with CA, and long-term low-dose administration of noradrenaline could lead to excessive myocardial oxygen consumption, which is associated with death or poor neurological outcomes. In this study, most patients treated with noradrenaline experienced hypotension or shock before and after developing IHCA. Noradrenaline had fewer side effects and a lower incidence of arrhythmia than dopamine (16), and its combination with dobutamine may be more effective and safer (24).

This study has some limitations. First, it was a single-center retrospective study using a small sample size. Second, the analysis was based solely on existing clinical data. Therefore, it is imperative to incorporate supplementary data, such as chest compression quality and specific laboratory indicators, in further studies. This will allow for a comprehensive examination of the clinical characteristics of patients with IHCA and the development of a predictive prognostic model. Consequently, further large-scale and multicenter prospective studies are required to corroborate the results of this study.

## 5 Conclusions

Most patients with IHCA who underwent CPR were older men. The etiology of IHCA was predominantly noncardiac, and the initial heart rhythm was mainly non-shockable. The continuous ROSC rate was 33.73%, and the good neurological prognosis rate was 6.82%.

Male sex, advanced age, CD >23 min, and TDOA >3 mg were identified as independent risk factors for non-ROSC. Independent protective factors identified before IHCA included arrhythmia, cardiac rhythm of initial defibrillation, and advanced airway intervention. The risk factors for PNO-12 were respiratory failure or shock, occurrence in a monitoring area, advanced airway intervention, and noradrenaline.

Cardiac etiology and initial defibrillation rhythm were identified as independent protective factors. Both models demonstrated good degrees of differentiation, fit, and clinical applicability, allowing for improved prediction of short- and long-term outcomes in patients with IHCA. These models can help clinicians provide more appropriate treatment measures and predict the prognosis for adult patients with IHCA.

## Data availability statement

The raw data supporting the conclusions of this article will be made available by the authors, without undue reservation.

## Ethics statement

The studies involving humans were approved by The First Hospital of Jilin University Institutional Review Board for studies involving humans (Date 2023/09/27, Num.2023-649). Written informed consent was not required to participate in this study in accordance with the local legislation and institutional requirements.

## Author contributions

ZL: Conceptualization, Data curation, Formal analysis, Investigation, Methodology, Project administration, Resources, Software, Writing – original draft, Writing – review & editing. JX: Conceptualization, Funding acquisition, Resources, Supervision, Validation, Visualization, Writing – review & editing.
