# Supplementary material for: A model for predicting return of spontaneous circulation and neurological outcomes in adults after in-hospital cardiac arrest: development and evaluation
Source: Front Neurol. 2023 Nov 17;14:1323721. doi: 10.3389/fneur.2023.1323721 (PMC10693474; doi:10.3389/fneur.2023.1323721)
Supplement: Supplementary file 1 [file Table_1.docx]

**A Model for Predicting Return of Spontaneous Circulation and Neurological Outcomes in Adults after In-Hospital Cardiac Arrest: Development and Evaluation**

**Supplementary table 1.** Characteristics, features, and interventions of IHCA events stratified by primary and secondary outcome

| **Categories** | **All patients n=851(%)** | **ROSC n=287(%)** | **Non-ROSC n=564(%)** | ***P* value^†^** | **CPC1-2 n=58(%)** | **CPC3-5 n=229(%)** | ***P* value^‡^** |
| --- | --- | --- | --- | --- | --- | --- | --- |
| **Sex** |  |  |  | ＜0.001 |  |  | 0.167 |
| Male | 543(63.81) | 160(55.75) | 383(67.91) |  | 37(63.79) | 123(53.71) |  |
| Female | 308(36.19) | 127(44.25) | 181(32.09) |  | 21(36.21) | 106(46.29) |  |
| **Age (years)** |  |  |  | 0.037 |  |  | 0.095 |
| ＜50 | 142(16.69) | 55(19.16) | 87(15.43) |  | 7(12.07) | 48(20.96) |  |
| 50-59 | 172(20.21) | 59(20.56) | 113(20.04) |  | 14(24.14) | 45(19.65) |  |
| 60-69 | 231(27.14) | 81(28.22) | 150(26.60) |  | 16(27.59) | 65(28.38) |  |
| 70-79 | 185(21.74) | 66(23.00) | 119(21.10) |  | 19(32.76) | 47(20.52) |  |
| ≥80 | 121(14.22) | 26(9.06) | 95(16.84) |  | 2(3.45) | 24(10.48) |  |
| M(IQR) | 65(54,74) | 64(53,72) | 66(55,75) | 0.022 | 63.75  (63.5,72) | 64（52.5,72） | 0.602 |
| **BMI (kg/m^2^)** | 22.69±2.59 | 22.57±2.26 | 22.75±2.74 | 0.703^a^ | 22.60±2.38 | 22.56±2.24 | 0.449^a^ |
| Normal weight | 586(68.86) | 202(70.37) | 384(68.08) |  | 40(68.97) | 162(70.74) |  |
| Underweight | 25(2.93) | 7(2.43) | 18(3.20) |  | 3(5.17) | 4(1.75) |  |
| Overweight | 230(27.03) | 76(26.50) | 154(27.30) |  | 15(25.86) | 61(26.64) |  |
| Obesity | 10(1.18) | 2(0.70) | 8(1.42) |  | 0(0.00) | 2(0.87) |  |
| **Comorbidities** |  |  |  |  |  |  |  |
| Hypertension | 343(40.31) | 113(39.37) | 230(40.78) | 0.692 | 23(39.66) | 90(39.30) | 0.961 |
| CHD | 272(31.96) | 107(37.28) | 165(29.26) | 0.018 | 36(62.07) | 71(31.00) | ＜0.001 |
| Heart failure | 161(18.92) | 66(23.00) | 95(16.84) | 0.030 | 19(32.76) | 47(20.52) | 0.048 |
| Arrhythmia | 222(26.09) | 95(33.10) | 127(22.52) | 0.001 | 31(53.45) | 64(27.95) | ＜0.001 |
| Pneumonia | 317(37.25) | 107(37.28) | 230(37.23) | 0.989 | 14(24.14) | 93(40.61) | 0.020 |
| Respiratory-F | 363(42.66) | 94(32.75) | 269(47.70) | ＜0.001 | 2(3.45) | 92(40.17) | ＜0.001 |
| Diabetes | 200(23.50) | 74(25.78) | 126(22.34) | 0.263 | 15(25.86) | 59(25.76) | 0.988 |
| Hepatic-I | 91(10.69) | 34(11.85) | 57(10.11) | 0.437 | 2(3.45) | 32(13.97) | 0.027 |
| Renal-I | 200(23.50) | 71(24.74) | 129(22.87) | 0.544 | 10(17.24) | 61(26.64) | 0.139 |
| Acute stroke | 146(17.16) | 38(13.24) | 108(19.15) | 0.031 | 2(3.45) | 36(15.72) | 0.014 |
| MCFD | 262(30.79) | 66(23.00) | 196(34.75) | ＜0.001 | 7(12.07) | 59(25.76) | 0.027 |
| Malignancy | 116(13.63) | 29(10.10) | 87(15.43) | 0.032 | 1(1.72) | 28(12.23) | 0.018 |
| MEA | 605(71.09) | 213(74.22) | 392(69.50) | 0.152 | 38(65.52) | 75(76.42) | 0.090 |
| Shock | 302(35.49) | 88(30.66) | 214(37.94) | 0.036 | 4(6.90) | 84(36.68) | ＜0.001 |
| **Presumed etiology** | |  |  | ＜0.001 |  |  | ＜0.001 |
| Cardiac | 354(41.60) | 144(50.17) | 210(37.23) |  | 52(89.66) | 92(40.17) |  |
| Non-cardiac | 497(58.40) | 143（49.83) | 354(62.77) |  | 6(10.34) | 137(59.83) |  |
| **IHCA location** |  |  |  | 0.362 |  |  | ＜0.001 |
| General area | 166(19.51) | 51(17.77) | 115(20.39) |  | 25(43.10) | 26(11.35) |  |
| Intensive area | 685(80.49) | 236(82.23) | 449(79.61) |  | 33(56.90) | 223(88.65) |  |
| **Initial rhythm** |  |  |  | ＜0.001 |  |  | ＜0.001 |
| Defibrillation | 141(16.57) | 95(33.10) | 46(8.16) |  | 43(74.14) | 52(22.71) |  |
| No-defibrillation | 710(83.43) | 192(66.90) | 518(91.84) |  | 15(25.86) | 177(77.29) |  |
| **Emergency measures** | |  |  |  |  |  |  |
| CD min, M(IQR) | 31(17,57) | 9(3,20) | 45(30,74) | ＜0.001 | 4.5(2,9.75) | 10(5,20) | ＜0.001 |
| Defibrillation | 203(23.85) | 107(37.28) | 96(17.02) | ＜0.001 | 43(74.14) | 64(27.95) | ＜0.001 |
| AAM | 448(70.04) | 134(46.09) | 314(55.67) | 0.013 | 6(10.34) | 128(55.90) | ＜0.001 |
| **Drugs administered** | |  |  |  |  |  |  |
| TDOA mg,M(IQR) | 3（1,7） | 1（0,2） | 5（2,9.75） | ＜0.001 | 0(0,0) | 1(0,3) | ＜0.001 |
| Norepinephrine | 290(34.08) | 87(30.31) | 203(35.99) | 0.098 | 8(13.79) | 79(34.50) | 0.002 |
| Dopamine | 449(52.76) | 118(41.11) | 331(58.69) | ＜0.001 | 13(22.41) | 105(45.85) | 0.001 |
| SB | 222(26.09) | 59(20.56) | 163(28.90) | 0.009 | 2(3.45) | 57(24.89) | 0.001 |
| Atropine | 269(31.61) | 61(21.25) | 208(36.88) | ＜0.001 | 4(6.90) | 57(24.89) | 0.003 |
| Lidocaine | 98(11.52) | 36(12.54) | 62(10.99) | 0.503 | 15(25.86) | 21(9.17) | 0.001 |

ROSC, return of spontaneous circulation; Non-ROSC, none-return of spontaneous circulation; †, primary outcome; ‡, secondary outcome; CPC, cerebral performance category; BMI, body mass index; CHD, coronary atherosclerotic heart disease; Respiratory-F, respiratory failure; Hepatic-I, hepatic insufficiency; Renal-I, renal insufficiency; MCFD, motor, cognitive or functional deficits; MEA, metabolic or electrolyte abnormality; CD, CPR duration; AAM, advanced airway management; TDOA, total dosage of adrenaline; SB, sodium bicarbonate ; a=Fisher’s exact probability
